# A Text-Book on the Practice of Medicine

**Published:** 1898-03

**Authors:** 


A TEXT-BOOK ON THE PRACTICE OF MEDICINE.—By James M. Anders, M. D., Ph. D., LL. D., Professor of the Practice of Medicine and of Clinical Medicine in the Medico-Chirurgical College; attending physician to the Medico-Chirurgical and Samaritan Hospitals, etc. Published by W. B. Saunders, 935 Walnut street, Philadelphia. Cloth, $5.00, net; sheep or half morocco, $6.50, net.

It has been a source of much pleasure as well as profit to read, for the purpose of review, this splendid work of 1287 pages, each separate page of which is replete with a full, yet concise, statement of all the facts connected with the subject. The work is profusely illustrated, colored plates being used whenever necessary, to elucidate the text.

The most valuable feature of the work is its compactness. No redundancy of language or irrelevant matter has been allowed to mar its pages, yet nothing has been left out. Neither has clearness of expression been sacrificed in the cause of brevity. Impractical theories and requirements of the laboratory have scant attention, and in the space usually alloted to them in such works, we have here the rich results of a lifetime experience at the bedside.

Under the head of etiology, bacteriology, so far as it is known, is clearly discussed, and what is of more consequence to the busy practitioner with a limited library, the best bacteriological methods are given.

A feature of especial value is the numerous diagnostic tables scattered throughout the work, most of them being original.

The subject of diagnosis, the keystone of medical knowledge, receives especial attention, and this feature alone would make the work invaluable.

The subject of malaria, of perennial interest to the Southern physician, is better treated than in any other work known to me. The author has evidently had a large personal experience in the treatment of its milder forms.

Clinically, the Southern practitioner finds himself at variance with the teachsngs of all the standard works on practice. In this work no reference is made to the use of calomel or any other cathartic in the treatment of any form of malaria, except bilious remittent fever, and this reference is accidental. Of course we all know that mercury in some form, or other purgative, is given almost universally. The writer was educated in a Southern college, and has a considerable acquaintance with the profession in three Southern States, and has never met a regular physician who did not pursue this practice. In the words of the distinguished Shoemaker, "No matter what may be the apparent results of laboratory experiments, they should not be given preference over the clinical experience of several generations of intelligent Southern physicians."

It yet remains for a representative work to be written by a Southern physician that will fairly reflect our experience.

On the whole it may be safely stated that this work is thoroughly representative of our present knowledge, and is indispensable to the doctor who has not read a recent work on practice. Several of the chapters deserve special mention, among them being that devoted to diseases of the heart, which is alone worth many times the price of the work.

Mechanically, the book is an excellent specimen of the printer's art. I. J. J.

